# Suppressor mutants demonstrate the metabolic plasticity of unsaturated fatty acid synthesis in *Pseudomonas aeruginosa* PAO1

**DOI:** 10.1099/mic.0.001400

**Published:** 2023-10-11

**Authors:** Huijuan Dong, John E. Cronan

**Affiliations:** ^1^​ Departments of Microbiology, University of Illinois at Urbana-Champaign, Urbana, Illinois, USA; ^2^​ Departments of Biochemistry, University of Illinois at Urbana-Champaign, Urbana, Illinois, USA

**Keywords:** desaturase, LptA, suppressors, Pseudomonas, desaturation

## Abstract

*
Pseudomonas aeruginosa* PAO1 has two aerobic pathways for synthesis of unsaturated fatty acids (UFAs), DesA and DesB plus the oxygen independent FabAB pathway. The DesA desaturase acts on saturated acyl chains of membrane phospholipid bilayers whereas the substrates of the DesB desaturase are thought to be long chain saturated acyl-CoA thioesters derived from exogeneous saturated fatty acids that are required to support DesB-dependent growth. Under suitable aerobic conditions either of these membrane-bound desaturates can support growth of *P. aeruginosa ∆fabA* strains lacking the oxygen independent FabAB pathway. We previously studied function of the *desA* desaturase of *P. putida* in a *P. aeruginosa ∆fabA ∆desA* strain that required supplementation with a UFA for growth and noted bypass suppression of the *P. aeruginosa ∆fabA ∆desA* strain that restored UFA synthesis. We report three genes encoding lipid metabolism proteins that give rise to suppressor strains that bypass loss of the DesA and oxygen independent FabAB pathways.

## Introduction

Biological membranes maintain appropriate fluidity to support membrane structure and function under various growth conditions. When adapting to an environment that requires greater membrane lipid rigidity, cells produce saturated fatty acids (SFA). In contrast, when membrane lipid function requires increased fluidity, more unsaturated fatty acids (UFAs) and/or branched-chain fatty acids are produced.

*Pseudomonas aeruginosa* is a Gram-negative opportunistic pathogen having membrane phospholipids composed of straight-chain SFAs and UFAs. In addition to the FabA-FabB oxygen-independent pathway, it has two oxygen-dependent desaturase pathways for UFA synthesis, DesA and DesB [1]. DesA introduces a double bond in the Δ9 position of 16 : 0 FA chains esterified to the *sn-*2 position of existing phospholipids. In contrast, DesB desaturates exogenously supplied saturated acyl chains probably following conversion to 16 : 0-CoA and 18 : 0-CoA to produce 16 : 1Δ9-CoA and 18 : 1Δ9-CoA, respectively [1]. The resulting unsaturated acyl-CoA are incorporated into phospholipids [2, 3]. Transcription of the *desCB* operon is regulated by the DesT repressor and acyl-CoA ligands. DesT binds the *fabAB* and *desCB* promoters [2, 3]. Saturated acyl-CoAs decrease the binding affinity of DesT to the *desCB* promoter thereby increasing desaturase levels and UFA production. In contrast unsaturated acyl-CoAs enhance DesT binding to the *fabAB and desCB* promoters to decrease UFA synthesis [4]. However, the levels of transcription repression differ, <3-fold and ~9-fold for *fabAB* and *desCB*, respectively [1, 2].

FA synthesis in both *P. aeruginosa* and *E. coli* results in acyl-ACP acyl chains of 16 or 18 carbons in length that are transferred to glycerol-3-phosphate to form phosphatidic acid, the key phospholipid precursor. The chains are transferred from acyl-ACP or acyl-CoA to the *sn*-1-position of glycerol-3-phosphate to form lysophosphatidic acid, LPA (formally 1-acyl-*sn-*glycerol-3-phosphate). In *E. coli* LPA is acylated in the *sn*-2-position to form phosphatidic acid by the essential PlsC LPA acyltransferase [5, 6]. However, many bacteria including pseudomonads encode multiple LPA acyltransferases that have only low amino acid sequence identity to PlsC, although active site and substrate binding residues are generally conserved.

Although we have a good understanding of the *E. coli* phosphatidic acid synthesis pathway, the *Pseudomonas* pathways remain largely unknown. As pointed out by Meredith and coworkers [7], ‘fatty acid metabolism is especially complex in pseudomonads, with an unusually large genetic allocation to both the catabolism and anabolism of fatty acids.’ These genomes encode multiple putative LPA acyltransferases, a few of which have been demonstrated to have LPA acyltransferase activity. However, unlike *E. coli* PlsC, no single LPA acyltransferase has been shown to be essential for growth.

A *P. aeruginosa* PAO1 strain lacking both the oxygen-independent pathway (*∆fabA*) and the DesA desaturase (*∆desA*) is a UFA auxotroph [1]. However, when plated without UFA supplementation, the *∆fabA ∆desA* strain suppressor colonies arose which restored UFA synthesis as well as growth in the absence of oleic acid. Sequencing of the genomes of four such suppressor strains showed mutations in three different genes.

## Construction of complementation strains

### Materials

DNA polymerase, restriction endonuclease, and T4 ligase were purchased from New England Biolabs. Sodium [1-^14^C]acetate (specific activity, 57.0 mCi mmol^−1^) and [1-^14^C]stearic acid (specific activity, 53.0 mCi mmol^−1^) were provided by Moravek, Inc. All the other reagents were of the highest available quality. Oligonucleotide primers were synthesized by Integrated DNA Technologies.

### Bacterial strains, plasmids, and growth conditions

The strains and plasmids used are given in (Table 1). *P. aeruginosa* strains were grown at 37 °C and *P. putida* strains were grown at 30 °C in Luria-Bertani (LB) medium containing (in g l^−1^) (tryptone, 10; yeast extract, 5; NaCl, 10; pH 7.0) When required, antibiotics and inducers were added as follows (in μg ml^−1^): gentamicin, 90; isopropyl-β-d-thiogalactoside (IPTG), 2 40. Xylose was added at 50 mM. Oleate was used at a final concentration of 0.5 mM. Growth was assayed by colony formation on solid media.

**Table 1.** Strains, plasmids and oligonucleotide primers

* P. putida* strain		
HJ25	Δ*fabA∆desA*	[23]
*** P. aeruginosa* PAO1 strains**		
PAO1	Wild-type	Lab Store
PA0682	*∆fabA∆desA*	[22]
HJ655	*∆fabA∆desA* suppressor 1	This work (Fig. 2)
HJ656	*∆fabA∆desA* suppressor 2	This work (Fig. 2)
HJ657	*∆fabA∆desA* suppressor 3	This work (Fig. 2)
HJ659	*∆fabA∆desA* suppressor 6	This work (Fig. 2)
**Plasmids**		
pSRK	Gm^r^, expression plasmid	Lab store
pMQ652	Gm^r^, Shuttle vector, pBBR1, with *xutR-Pxut* and multicloning site.	[8]
pHJ85	*P. aeruginosa desB* expression plasmid derived from pMQ652	This work
pHJ177	*P. aeruginosa desCB* expression plasmid derived from pMQ652	This work
pHJ312	*P. aeruginosa acdA* expression plasmid derived from pSRKGm	This work
pHJ12	*P. aeruginosa lptA* expression plasmid derived from pSRK Gm^r^	This work
pHJ100	*P. aeruginosa lptA* (224 aa up deletion) expression plasmid derived from pSRK Gm^r^	This work
pHJ101	*P. aeruginosa lptA* (224 aa down deletion) expression plasmid derived from pSRK Gm^r^	This work
pHJ13	*P. aeruginosa acdA* expression plasmid derived from pSRK Gm^r^	This work
pHJ45	*P. aeruginosa desT* expression plasmid derived from pSRK Gm^r^	This work
Oligonucleotide primers		
Primer^∗^	Sequence 5′-3′	
Pa0506(*acdA*) *Nde*I up	GGAATTCcatATGCCTGATTACAAGGCCC	
Pa0506 *Hind*III dn	CCCaagcttTCAGTAACCGAGGGCGAAA	
Pa0005(*lptA*) *Nde*I up	GGAATTCcatATGTCGACAGTGCAGGC	
Pa0005 *Hin*dIII dn	CCCaagcttTCACGAGACCACCGACG	
Pa0005 *Nde*I up(224 aa)	GGAATTCcatATGCTGCCGTTCCGCGC	
Pa0005 *Hind*III dn(224 aa)	CCCaagcttCTAGGCGATGGCGCG	
Pa4890(*desT*) *Nde*I up	GGAATTCcatATGTCCTCACCCCGCGC	
Pa4890 *Hind*III dn	CCCaagcttTCAGCCGGGCAGGCC	
Pa*desB Bam*HI up	CGGGATCCATGAACCGACCTGCGAGC	
Pa*desCB Bam*HI up	CGGGATCCATGAACCGACCTGCGAGC	
Pa*desCB Hin*dIII dn	CCC*aagctt*CGGCATTATCCCCGCTC	

*The underlines indicate restriction enzyme sites.

LPA, lysophosphatidic acid.

### Materials

DNA polymerase, restriction endonuclease, and T4 ligase were purchased from New England Biolabs. Sodium [1-^14^C]acetate (specific activity, 57.0 mCi mmol^−1^) and [1-^14^C]stearic acid (specific activity, 53.0 mCi mmol^−1^) were provided by Moravek, Inc. All the other reagents were of the highest available quality. Oligonucleotide primers were synthesized by Integrated DNA Technologies.

### Bacterial strains, plasmids, and growth conditions

The strains and plasmids used are given in Table 1. *P. aeruginosa* strains were grown at 37 °C and *P. putida* strains were grown at 30 °C in Luria-Bertani (LB) medium containing (in g l^−1^) (tryptone, 10; yeast extract, 5; NaCl, 10; pH 7.0) When required, antibiotics and inducers were added as follows (in μg ml^−1^): gentamicin, 90; isopropyl-β-d-thiogalactoside (IPTG), 2 40. Xylose was added at 50 mM. Oleate was used at a final concentration of 0.5 mM. Growth was assayed by colony formation on solid media.

### Construction of complementation strains

The targeted gene fragment was amplified by PCR and the primers are given in Table 1, *PadesB* and *PadesCB* were digested with BamHI and HindII and ligated into the pMQ652 vector [8] digested with the same enzymes whereas *lptA* and its deletion derivatives, *acdA* and *desT* were digested with NdeI and HindIII, and ligated into vector pSRKGm [9] digested with the same enzyme. After screening and verification, the plasmids were transferred to the host cell by electroporation. For *Pseudomonas *, electroporation was performed as follows: a 5 ml culture of the host strain was grown overnight, 1 ml of the host strain culture was washed twice with 500 mM sucrose, concentrated to 100 µl, the complementation plasmid was added. The solution was transferred to the electroporation cuvette and put on ice for 10 min, after a 2.5 KV shock, 1 ml of fresh medium was added, the cultures were grown for 1 h at 37 °C (* P. aeruginosa *) or 30 °C (* P. putida *) and plated on media containing the appropriate selective antibiotic.

### DNA sequencing

Genomic sequencing (Oxford Nanopore confirmed by Illumina sequencing) was done by the DNA Services Laboratory of the Carver Biotechnology Centre on this campus. Genomic DNAs was purified using the Promega DNA Purification Kit. Briefly, 1.5 ml of overnight culture strains were harvested and the bacterial genomic DNAs were purified according to the kit. Finally, DNA concentrations were determined by absorbance and DNA quality was checked by agarose gel electrophoresis.

### Thin Layer Chromatography (TLC) analysis of phospholipid acyl chains

*P. aeruginosa* and *P. putida* strains were grown to OD_600_ 0.5 with 0.5 mM oleic acid, the cells were harvested by centrifugation, washed three times and incubated for another 3 h at 37 °C (* P. aeruginosa *) in the presence of [1-^14^C]acetate (final concentration of 1 µCi ml^−1^) or [1-^14^C]stearic acid (final concentration of 0.1 µCi ml^−1^). Cultures were lysed with methanol-chloroform (2 : 1). The phospholipids were extracted with chloroform and dried under nitrogen. The fatty acyl groups on phospholipids were then converted to their methyl esters by transesterification with 25 % sodium methoxide, extracted into petroleum ether, taken to dryness under nitrogen, resuspended in hexanes and loaded onto 20 % silver nitrate TLC plates (Analtech) which were developed in toluene at −20°C. Inclusion of silver allows separation of saturated and unsaturated esters and low temperature development allows separation of double bond isomers. The plates containing the [1-^14^C]-labelled esters were analysed by phosphorimaging using a GE Typhoon FLA700 Scanner and analysed by the Image Quant TL programme.

## Results

### Growth of the suppressor strains

**Fig. 1.**
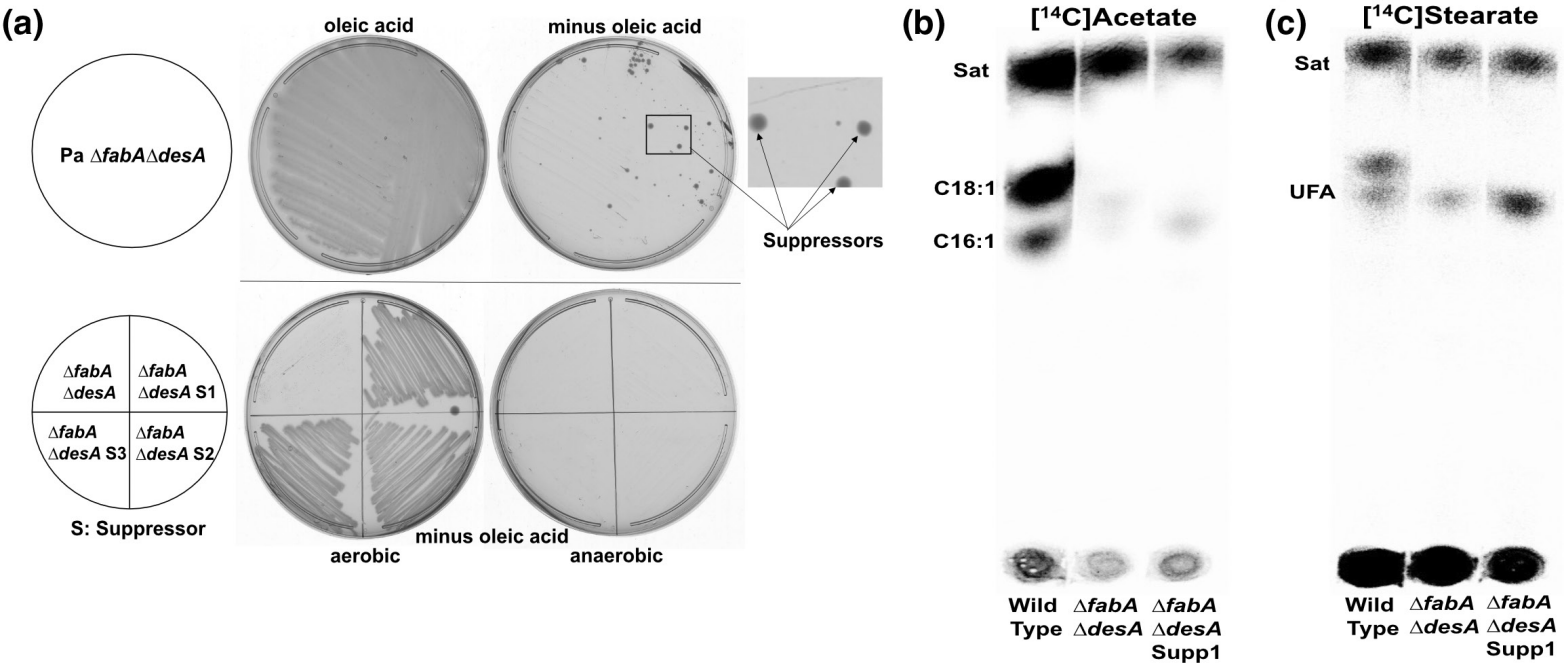
Suppressors of the *P. aeruginosa* PAO1 *∆fabA ∆desA* strain. (**a)** Suppressor colonies arose on LB medium without oleic acid and grew well upon restreaking on the same medium but only under aerobic conditions. Labelling of suppressor strain one cultures with [1-^14^C]acetate (**b**) or [1-^14^C]stearic acid (**c**) showed UFA synthesis was restored in the suppressor strain. The other three suppressors showed similar growth and in the absence of oleic acid. UFA synthesis was also restored in the other three suppressors as seen for suppressor S2 in Fig. 4.

The suppressor strains required aerobic conditions for growth indicating that bypass of the *∆fabA ∆desA* mutations is due to desaturation (Fig. 1a). Since *P. aeruginosa* has only two desaturases DesA and DesB [1] and DesA was eliminated by the *∆desA* deletion, aerobic growth of the suppressor strains must involve DesB. Indeed, labelling of cultures with [^14^C]acetate (Fig. 1b) or [^14^C]stearate (Fig. 1c and data below) showed that all four suppressor strains synthesize ∆9 UFA species which are the products of DesB, the sole desaturase of the *∆fabA ∆desA* strain. We used [^14^C]stearate rather than [^14^C]palmitate to directly assay DesB activity because desaturation of stearate gives the ∆9 C18 species which can readily be distinguished from the ∆11 C18 species formed by *de novo* synthesis whereas both palmitate desaturation and *de novo* synthesis produce ∆9 C16.

### Genome sequences of the suppressor strains and the effects of their mutations on growth

**Fig. 2.**
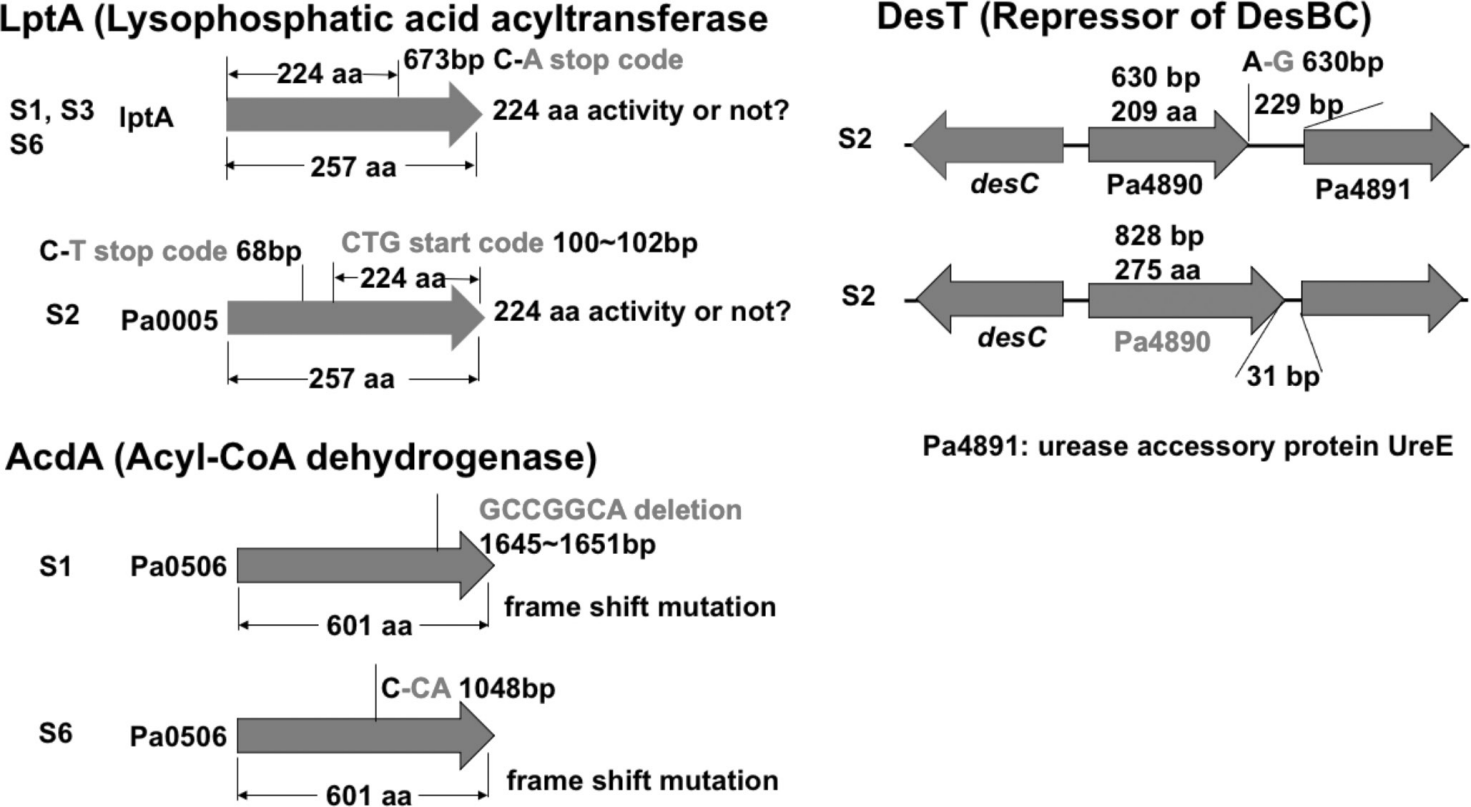
The mutations present in the suppressor strains. All four strains have a mutation in *lptA* (PA0005). Deletion mutations were found in *acdA* (PA0506) encoding an acyl-CoA dehydrogenase in suppressor strains S1, S3 and S6 whereas suppressor S2 also contained a mutation in *desT* (PA4890) the repressor of *desB* expression. Our work focused on suppressor two because it contains the two best-defined mutations in the *lptA* and *desT* genes of those altered in the suppressor strains.

The four suppressor strains were submitted for whole genome sequencing (Oxford Nanopore confirmed by Illumina sequencing). All suppressor strains retained the *∆fabA* and *∆desA* deletion alleles (Fig. 2). Strikingly, all four suppressor strains contained mutations in *lptA* (PA0005) which encodes an LPA acyltransferase that complements growth of the *E. coli plsC*(Ts) strain at the nonpermissive temperature [10]. In three of the four suppressor strains (S1, S3 and S6) the *lptA* mutation is a premature stop codon that encodes a protein truncated by loss of 33 C-terminal residues whereas suppressor S2 has a different *lptA* mutation. The suppressor S2 LptA protein lacks the first 23 residues which would remove most of the hydrophobic helix one required for membrane binding and activity as shown in the *Thermatoga maritima* LPA [11]. Moreover, suppressor S2 has a mutation in *desT,* the transcriptional repressor of *desB* . Two suppressor strains, S1 and S6, also contain a mutation in an acyl-CoA dehydrogenase (PA0506) that we have named AcdA. We believe that AcdA is a bona fide acyl-CoA dehydrogenase because it is 91 % identical to *P *. *putida* KT2440 dehydrogenase (PP_0368) where activity of the purified protein has been demonstrated [12].

### Effects of the suppressor mutations and complementation by wild-type alleles

**Fig. 3.**
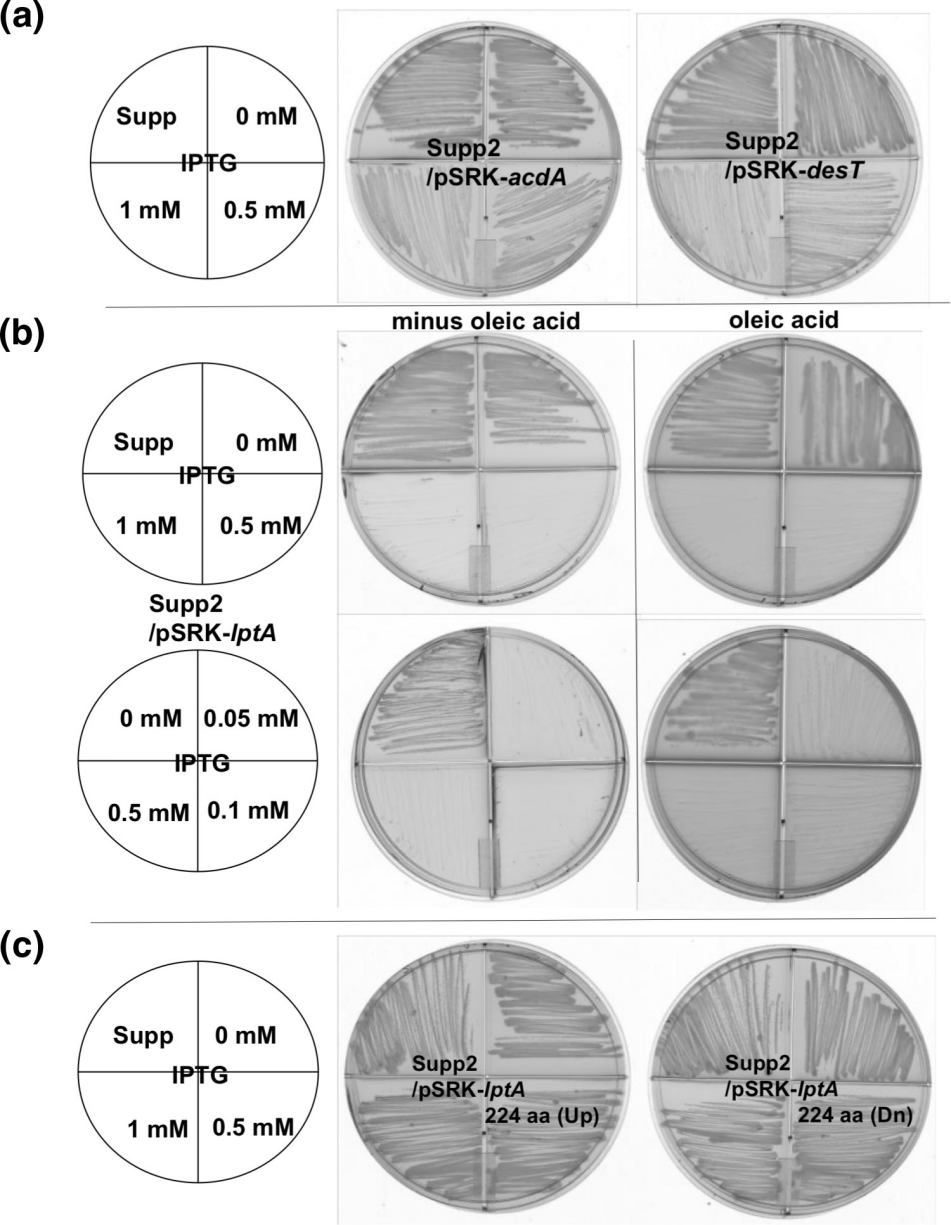
Inhibition of the growth of suppressor two (Supp) by expression of the wild-type copies of the mutant genes. (**a)** Expression of either the AcdA acyl-CoA dehydrogenase or the DesT repressor inhibited growth of the suppressor strain two upon induction of expression of the wild-type gene. (**b)** Growth of suppressor strain two was completely blocked upon induction of wild-type *lptA*. Addition of oleic acid only very weakly supported growth. (**c)** The deleted *lptA* alleles, unlike the wild-type gene, fail to block growth of suppressor strain two. Up and Dn refer to the upper and lower *lptA* mutants as depicted in Fig. 2. These data indicate that all mutant alleles of suppressor strain two encode proteins that lack function.

The mutations of the suppressor strains could be loss of function or gain of function alleles (albeit much less likely for deletions). This was tested by expression of the wild-type of each mutant allele from a tightly regulated promoter. Expression of the wild-type gene in each of the suppressor strains decreased or blocked both growth and UFA synthesis indicating that the suppressor mutations are loss of function. Expression of the wild-type DesT in suppressor two decreased growth and UFA synthesis even in the absence of induction indicating that the DesT of suppressor two is inactive (Fig. 3a). Similar results were seen upon expression of the wild-type AcdA (Fig. 3a). The most dramatic effect was that seen upon introduction of a plasmid encoding wild-type LptA into suppressor two. Induction of wild-type LptA expression resulted in complete blockage of growth and UFA synthesis (Fig. 3b) whereas the *lptA* deletion alleles (Fig. 2) did not block growth (Fig. 3c). These data also showed that the plasmids that expressed the wild-type *lptA* allele almost eliminated suppressor accumulation suggesting that routes to *∆fabA ∆desA* suppression that do not involve LptA inactivation are rare.

**Fig. 4.**
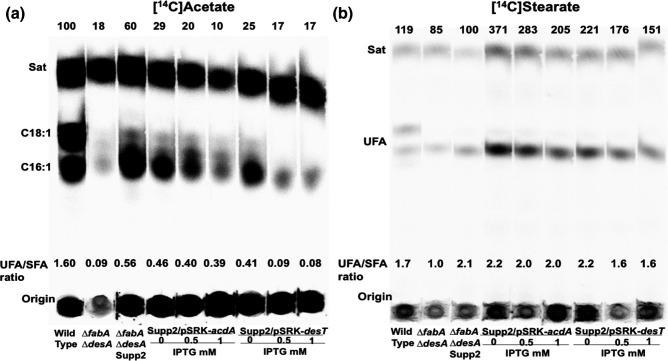
Effects of expression of the wild-type copies of *acdA* or *desT* on UFA synthesis. (**a)** Labelling with [^14^C]acetate. (**b)** Labelling with [^14^C]stearate. Expression was driven by the *lac* promoter of vector pSRKGm [9].

**Fig. 5.**
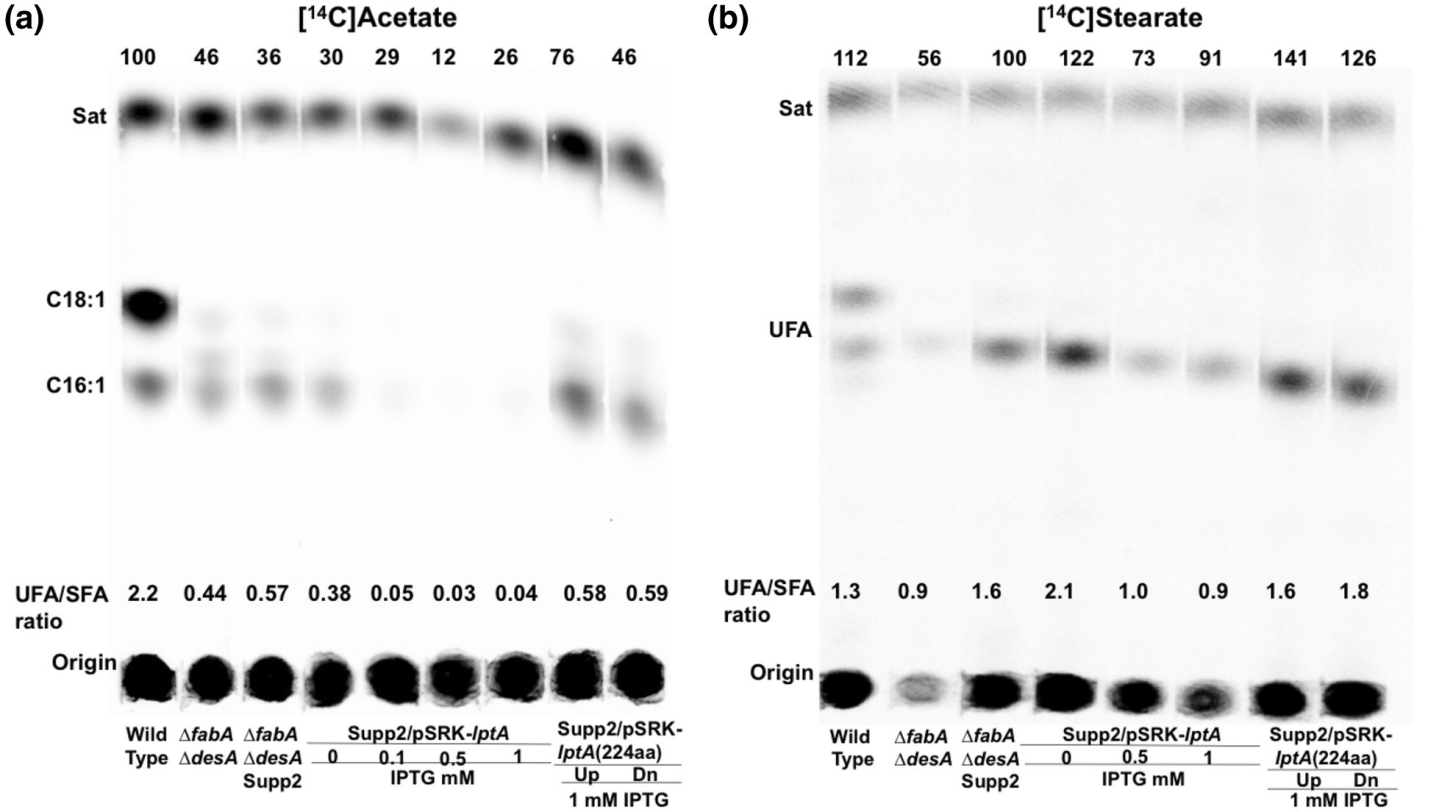
Effects of expression of the wild-type copy of *lptA* or deletion derivatives of *lptA* on UFA synthesis. (a) Labelling with [^14^C]acetate. (**b)** Labelling with [^14^C]stearate. Expression was driven by the *lac* promoter of vector pSRKGm [9].

The effects of the wild-type alleles on UFA synthesis were assayed by labelling of cultures with either [^14^C]acetate or [^14^C]stearate (Figs 4 and 5). Assay of *de novo* synthesis by [^14^C]acetate labelling showed decreased levels of UFA synthesis when wild-type copies of *desT* or *acdA* were introduced into suppressor two and the levels of UFA synthesis decreased with induction (Fig. 4a). As expected from the effects of expression of wild-type LptA on growth of suppressor two, induction of wild-type LptA expression abolished UFA synthesis (Fig. 5a). However, expression of either of the two *lptA* deletion alleles had no effect on UFA synthesis indicating that both are loss of function mutations (Fig. 5b). Direct assay of DesB desaturation by [^14^C]stearate labelling (Figs 4b and 5b) showed more modest effects, although induction of expression of the DesT wild-type allele decreased UFA formation as expected from increased repressor levels.

### DesB requires DesC for activity

**Fig. 6.**
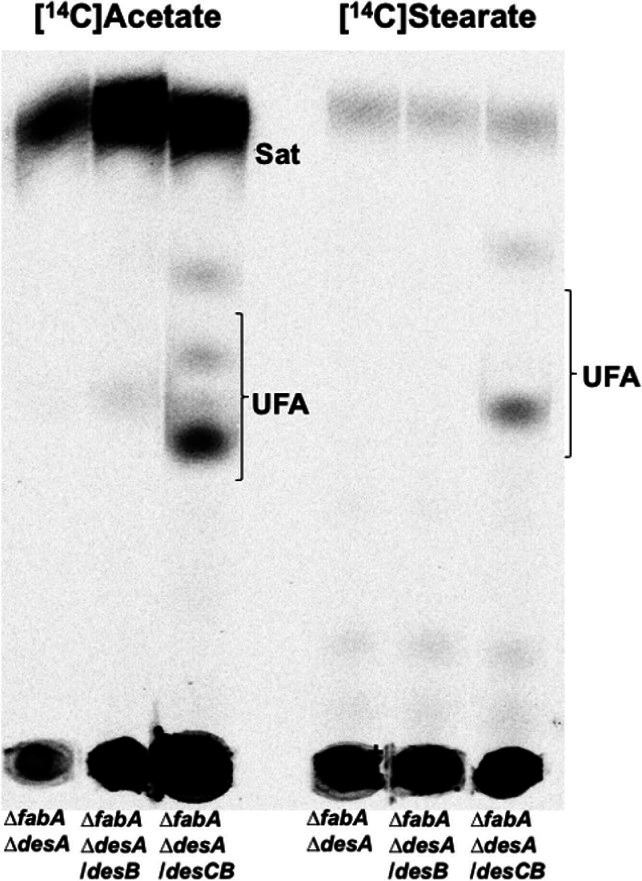
Desaturation by DesB requires DesC. The *P. putida ∆fabA ∆desA* strain [13] was transformed with plasmids encoding either *desB* or *desCB*. The left panel is [^14^C]acetate labelling whereas the right panel is [^14^C]stearate labelling. The genes were expressed from the xylose inducible promoter of vector pMQ652 [8] in the presence of 50 mM xylose.

Although we have interpreted desaturation in terms of DesB, DesB may not act alone. The *desB* gene is cotranscribed with *desC*, the product of which has been assigned an electron transfer-oxidoreductase role in the DesB desaturation reaction [1]. However, the proposed DesC requirement for DesB function has not been tested. The *P. putida* F1 *∆fabA ∆desA* strain provided a test because *P. putida* F1 lacks *desCB* [13]. DesB was expressed alone or together with DesC in the absence of the oleic acid required by the *∆fabA ∆desA* strain. Labelling with either [^14^C]acetate or [^14^C]stearate detected UFA synthesis only when both DesB and DesC were expressed, DesB alone was inactive (Fig. 6). These data indicate that *P. putida* F1 lacks a protein able to replace DesC. We tested the possibility that DesC might provide electron transfer-oxidoreductase function for *P. putida* F1 DesA but this was not the case. The *P. putida* F1 DesA expressing DesC remained unable to support growth of a *∆fabA* strain (data not shown).

## Discussion

All four suppressor strains have a loss of function mutation in *lptA*. Expression of *P. aeruginosa* PAO1 *lptA* is known to complement growth of the *E. coli plsC*(Ts) strain demonstrating that it is a functional LPA acyltransferase [10]. The *P. aeruginosa* PAO1 *lptA* deletion strain grew somewhat more slowly than the parent at 37 °C but much more poorly than the wild-type parent at 11 °C [10]. The fatty acid composition of the *∆lptA* strain differed somewhat from that of the wild-type strain in distribution between C16 and C18 chains. Complementation of the *∆lptA* strain with a plasmid expressing wild-type LptA restored the wild-type composition [10]. In *P. fluorescens* two LPA acyltransferase genes called *hdtS* and *patB* were also found to complement growth of the *E. coli plsC*(Ts) strain [14]. HdtS is 70 % identical to *P. aeruginosa* PAO1 LptA whereas there is no convincing *P. aeruginosa* PAO1 PatB homolog. A ∆*hdtS* strain showed a modest growth defect at 30 °C that was corrected by expression of *E. coli* PlsC whereas the ∆*patB* strain grew at 30 °C but poorly at 38 °C [14]. Note that *P. aeruginosa* PAO1 and *P. fluorescens* each have several annotated LPA acyltransferase genes in addition to those studied: five and six, respectively (www.microbesonline.org). Currently no single *Pseudomonas* LPA acyltransferase has been shown to be essential for growth.

A more complex situation exists for the *acdA* mutations of suppressor strains S1 and S6 because *P. aeruginosa* PAO1 encodes at least ten, perhaps as many as twenty acyl-CoA dehydrogenases. This multiplicity is consistent with the ability of *P. aeruginosa* PAO1 to grow on a large variety of aromatic and aliphatic compounds as carbon and energy sources. The rings of the aromatic compounds are cleaved to give aliphatic compounds which together with the aliphatic compounds are converted to acids and degraded by β-oxidation-like pathways [15]. However, the *Pseudomonas* acyl-CoA dehydrogenases have overlapping specificities which complicates assigning distinct metabolic roles. This is illustrated by a study in *P. putida* KT2440 which is thought to encode twenty-one acyl-CoA dehydrogenases [16]. Deletion derivatives of four of these genes were constructed and growth of the resulting strains on a range of alkanoic acids from propionic (C3) to palmitic (C16) acids was measured. All four deletion strains grew on all sixteen acids. Only one deletion strain had a phenotype and that was a modestly decreased ability to utilize C3-C9 acids [16].

The only mutation of a suppressor strain that lacks putative homologs is the *desT* mutation of suppressor two which encodes a defective protein. DesT is a repressor that regulates the DesB and FabAB pathways [2]. (Only DesB is regulated in these strains due to the *fabA* deletion.) The *desT* mutation of suppressor two removed the *desT* termination codon resulting in a 66-residue extension of the protein. AlphaFold [17] predicts that the extension is essentially unstructured although some models contain a short (three turn) helix. The most likely consequence of the extension is inhibition of the DesT dimerization required for tight promoter binding [1, 4]. This would result in derepression of *desB* and an increased level of desaturase. As expected, overproduction of wild-type DesT repressed desaturation in suppressor two as measured by [^14^C]stearate labelling (Fig. 4b).

**Fig. 7.**
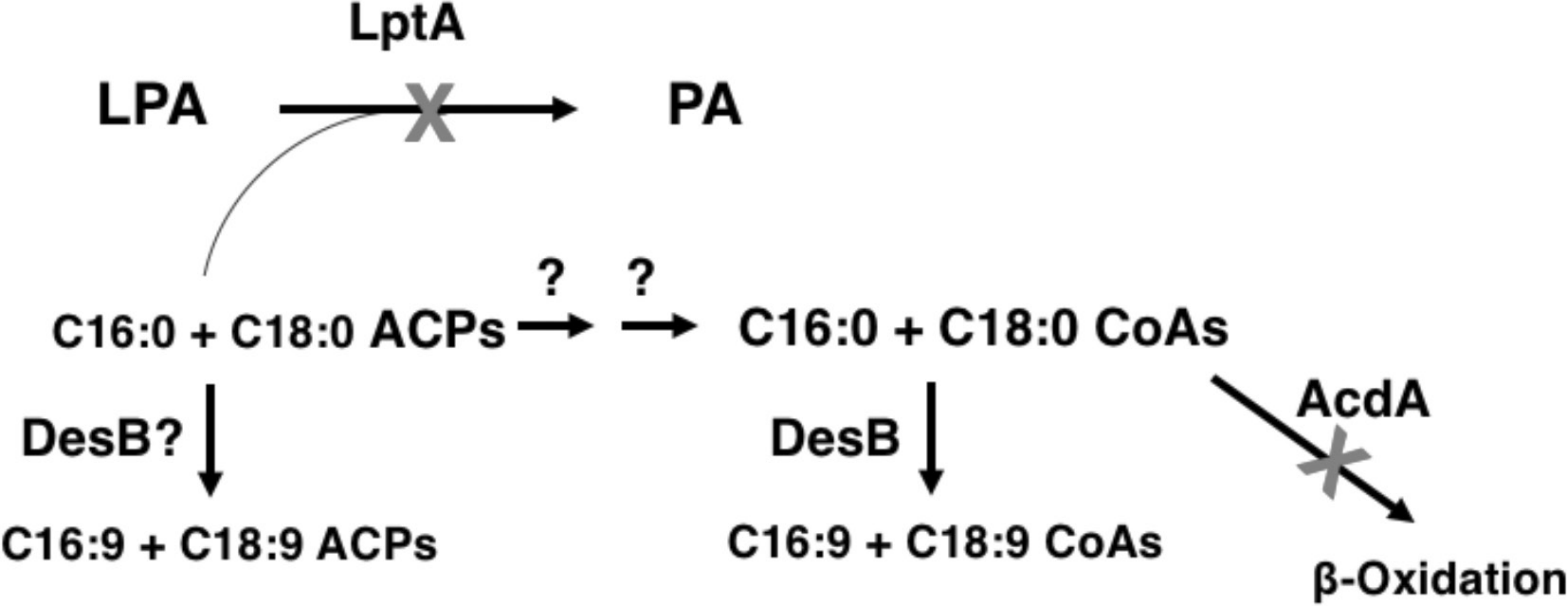
Current model of suppression of the *P. aeruginosa ∆fabA ∆desA* strain. All suppressor strains encode a nonfunctional LptA lysophospholipid acyltransferase. LptA inactivation is the key mutation and is proposed to result in the accumulation of the saturated acyl ACP species, C16 : 0 and C18 : 0. These could be DesB substrates or be converted to C16 : 0 and C18 : 0 CoA species. Conversion could be by several pathways, transacylation or less likely by cleavage by an acyl-ACP specific thioesterase to free fatty acids which would be converted to acyl-CoAs by an acyl-CoA synthetase. The role of DesT inactivation would be to increase DesB desaturase levels whereas the role of AcdA inactivation would be to prevent degradation of the putative acyl-CoA DesB substrates by the β-oxidation pathway.

A model (Fig. 7) based on these data that could explain suppression of the *∆fabA ∆desA* mutations is that the *lptA* mutations result in accumulation of C16 and C18 saturated acyl-ACPs and these are desaturated either directly or indirectly by DesB before incorporation into phospholipid. Soluble plant desaturases act on stearoyl-ACP and thus acyl-ACPs are plausible DesB substrates [18]. Another possibility is that the saturated acyl-ACPs could be transacylated to acyl-CoAs, a reaction catalysed by 3-ketoacyl-ACP synthases [19, 20] and are subsequently desaturated. (Although reasonable, there is no direct evidence that acyl-CoAs are DesB substrates.) In the model the role of the other mutations present in the suppressor strains is to optimize the effects of the LptA mutations. A secondary effect is consistent with the weaker effects of complementation with the wild-type alleles of *acdA* and *desT* mutations on growth and UFA synthesis. The *acdA* acyl-CoA dehydrogenase mutations would prevent degradation of the accumulated acyl-ACPs either directly or after their conversion to acyl-CoAs. The *desT* lesion of suppressor two would result in an increase in DesB desaturase levels.

The multiple annotated LPA acyltransferase and acyl-CoA dehydrogenase genes of *P. aeruginosa* PAO1 are a puzzle. What is special about LptA and AcdA? Why were these genes inactivated rather than genes encoding putative orthologs? The *P. aeruginosa* PAO1 *∆lptA* strain has a growth deficiency and an altered fatty acid composition (increased C18 chains at the expense of C16 chains) [10] whereas the only other LPA acyltransferase deletion strain, *∆patB* of *P *. *fluorescens *, does not [14]. The slow growth reported for the *P. aeruginosa* PAO1 *∆lptA* strain argues that strains lacking LptA may accumulate acyl-ACPs because LPA acyltransferase activity is limiting despite the other putative *P. aeruginosa* PAO1 LPA acyltransferases. There seems no straightforward explanation for multiple LPA acyltransferases, although at least one other acyltransferase must be active in the *P. aeruginosa* PAO1 *∆lptA* strain since LptA is not essential [10]. The phospholipid acyl chain composition of *P. aeruginosa* PAO1 is essentially the same as that of *E. coli* which has only a single LPA acyltransferase, PlsC. Hence, it seems unlikely that pseudomonads would need an array of acyltransferases for attachment of specific acyl chains. The *P. putida* acyl-CoA dehydrogenase (PP_0368) that is 91 % identical to *P. aeruginosa* PAO1 AcdA is significantly more active on palmitoyl-CoA and stearoyl-CoA than on any of fourteen other substrates tested [12]. Hence, the substrate specificity of AcdA may have provided a selection for its inactivation.

Another puzzle is that unlike *P. aeruginosa, desB* is an essential gene in *Acinetobacter baumannii,* a related obligately aerobic bacterium. *A. baumannii* is an unusual bacterium that lacks a *de novo* UFA synthesis pathway and must obtain UFA by desaturation. The genome is annotated as encoding eight desaturases, one of which, DesB, is essential for more than trace growth in the absence of UFA supplementation [21]. The *A. baumannii desB* is found in the same genome context as in *P. aeruginosa* and is regulated by a DesT homologue. *A. baumannii* also encodes a DesA desaturase which is not essential [21]. These phenotypes are the opposite of those in *P. aeruginosa* where in the absence of UFA synthesis (a *∆fabA* strain) *desA* is essential whereas *desB* is not [22]. The *A. baumannii* DesA seems weaker (or more poorly expressed) than *P. aeruginosa* DesA because a *∆desB* strain has less than one-third the UFA content of the parental strain and grows very poorly in the absence of UFA supplementation [21]. DesA is responsible for this residual UFA production because a *∆desA ∆desB* strain perishes in the absence of UFA supplementation [21]. Both *P. aeruginosa* and *A. baumannii* are Pseudomonadales isolated from human wounds and thus the switching of the physiological roles of the DesA and DesB in the two bacterial species is difficult to rationalize.
